# Predicting the stereoselectivity of chemical reactions by composite machine learning method

**DOI:** 10.1038/s41598-024-62158-0

**Published:** 2024-05-27

**Authors:** Jihoon Chung, Justin Li, Amirul Islam Saimon, Pengyu Hong, Zhenyu Kong

**Affiliations:** 1 Department of Industrial Engineering, Pusan National University, Busan, Korea; 2 Management, Entrepreneurship, and Technology, University of California, Berkeley, CA USA; 3 Grado Department of Industrial and Systems Engineering, Virginia Tech, Blacksburg, VA USA; 4 Department of Computer Science, Brandeis University, Waltham, MA USA

**Keywords:** Chemical engineering, Cheminformatics

## Abstract

Stereoselective reactions have played a vital role in the emergence of life, evolution, human biology, and medicine. However, for a long time, most industrial and academic efforts followed a trial-and-error approach for asymmetric synthesis in stereoselective reactions. In addition, most previous studies have been qualitatively focused on the influence of steric and electronic effects on stereoselective reactions. Therefore, quantitatively understanding the stereoselectivity of a given chemical reaction is extremely difficult. As proof of principle, this paper develops a novel composite machine learning method for quantitatively predicting the enantioselectivity representing the degree to which one enantiomer is preferentially produced from the reactions. Specifically, machine learning methods that are widely used in data analytics, including Random Forest, Support Vector Regression, and LASSO, are utilized. In addition, the Bayesian optimization and permutation importance tests are provided for an in-depth understanding of reactions and accurate prediction. Finally, the proposed composite method approximates the key features of the available reactions by using Gaussian mixture models, which provide suitable machine learning methods for new reactions. The case studies using the real stereoselective reactions show that the proposed method is effective and provides a solid foundation for further application to other chemical reactions.

## Introduction

Stereochemistry plays a critical role in the field of biology, where many biochemical processes in living cells rely on selective or specific reactions controlled by spatial molecular arrangement. In medicine, the significance of stereochemistry is well recognized, particularly regarding the effects of drugs. For example, single-stereoisomer formulations improve therapeutic indices for some therapeutics because they exhibit greater selectivity for their biological targets and/or better pharmacokinetics than a mixture of stereoisomers. In addition, different stereoisomers might also have contradictory effects on the human body. Specifically, one stereoisomer may have positive effects on the body. In contrast, another one may be less effective (D-Isoproterenol vs. L-Isoproterenol on the blood pressure or heart rate), ineffective (as in the case of the R enantiomer of ibuprofen), or even toxic (as in the case of thalidomide)^1^.

The stereoselectivity of a chemical transformation is an important and intriguing aspect of stereochemistry. It is the ability to control the formation of preferred specific stereoisomers during chemical reactions. The degree of this ability is highly dictated by the choice of reactants, catalysts, and other reaction conditions. Therefore, a quantitative understanding of the stereoselectivity of a chemical transformation is of great importance in organic synthesis. Unfortunately, we have only a limited and qualitative understanding. Though we understand that stereoselectivity arises from differences in steric and electronic effects in the mechanistic pathways, it is still frustrating to quantitatively rationalize or even predict stereoselectivity.

Enantioselectivity ($\Delta \Delta \text {G}^{\ddag }$) refers to the degree to which one enantiomer, a subtype of stereoisomer, is preferentially produced from the stereoselective reactions. Specifically, $\Delta \Delta \text {G}^{\ddag }$ equals to $-RT\ln {(\text {e.r.})}$, where e.r. is the enantiomeric ratio, *T* is the temperature at which the reaction was performed, and *R* is the gas constant^2^. Therefore, the extremely large or small value of $\Delta \Delta \text {G}^{\ddag }$ represents that a single enantiomer is dominantly produced from the reaction. Thus, predicting $\Delta \Delta \text {G}^{\ddag }$ is useful in various fields, including safer and more effective drug development. Furthermore, the prediction of the enantioselectivity can provide an improved quantitative understanding of the stereoselective reaction. To achieve this objective, this paper aims to build a model to predict the enantioselectivity of a stereoselective reaction for varying combinations of reaction conditions (reactants, solvents, catalysts, and other influential components).

Recently, machine learning techniques have been increasingly used in various application areas for quantitative analysis because of their superior performance. Regarding the prediction of the enantioselectivity, Reid et al.^2^ applied linear regression models to predict the enantioselectivity of the chiral phosphoric acid (CPA) reactions which are the representative stereoselective reactions^3,4^. Although linear regression models are straightforward to interpret, they fail to capture complex relationships between features, such as nonlinearity and interactions, hindering the accurate prediction of enantioselectivity. Moon et al.^5^ developed a machine learning model by using the Random Forest (RF) algorithm to predict the stereoselectivity in glycosylation reactions. Yu^6^ developed predictive models using Support Vector Machine (SVM) and RF algorithms to predict enantioselectivities in asymmetric catalytic reactions with a particular focus on thiol addition to N-acylimines catalyzed by chiral phosphoric acids. These models outperform traditional linear regression methods, which indicate the effectiveness of nonlinear machine learning algorithms in predicting enantioselectivities. Gao et al.^7^ developed predictive models by employing a wide range of machine learning techniques, including LASSO regression, SVM, k-Nearest Neighbors, Decision Trees, RF, XGBoost, and AdaBoost to predict the enantioselectivities of asymmetric phenolic dearomatization reactions. They employed a five-fold cross validation approach during model training for each algorithm and achieved optimal performance with the XGBoost algorithm. Recently, deep learning models have also been used for enantioselectivity prediction. Hoque et al.^8^ used a Deep Neural Network (DNN) to predict the enantioselectivity of catalytic asymmetric $\beta$-C-H bond activation reactions. Similarly, Hong et al.^9^ used DNN to predict the enantioselectivity of compounds in chiral chromatography based on their 3D conformations. In asymmetric reactions, machine learning methods are also widely used to accurately predict the Gibbs free energy since Gibbs free energy quantifies the energy difference between transition states leading to the formation of enantiomers. Specifically, Ferraz-Caetano et al.^10^ used RF, Gradient Boosting, SVM, and Multi-Layer Perceptron Neural Network techniques for predicting solvation Gibbs energy using open-source chemical features. Ward et al.^11^ developed a message-passing neural network model and trained it using a newly introduced dataset consisting of solvation energies for over 130,000 molecules in five solvents. Low et al.^12^ used Graph Neural Network (GNN) architecture for predicting solvation Gibbs free energy of molecules in different solvents where they incorporated chemically intuitive parameters such as partial atomic charges and solvent dielectric constant into the featurization process. Like the research of Low et al.^12^, some other work, such as Lim and Jung^13^ and Pathak et al.^14^ are also available in the literature that used GNN architectures for predicting solvation energy from pairwise atomistic interactions. All the machine learning models mentioned showed better accuracy in predicting Gibbs free energy than the traditional quantum mechanical methods. However, none of the work focuses on systematically providing appropriate machine learning methods based on the features of reactions for accurate prediction.

To address this reasearch gap, this work proposes a novel composite machine learning method. Specifically, machine learning methods that can capture the nonlinearity and interactions between features for accurate prediction are used in this work. In addition, the proposed composite machine learning methods choose the appropriate machine learning methods based on the feature characteristics of reactions to achieve the accurate prediction of enantioselectivity. Furthermore, some advanced data analytics techniques related to hyperparameter optimization and sensitivity analysis are provided for an in-depth understanding of reactions. The experimental results on a set of real stereoselective reactions show the effectiveness of our method.

## Data

**Figure 1:**
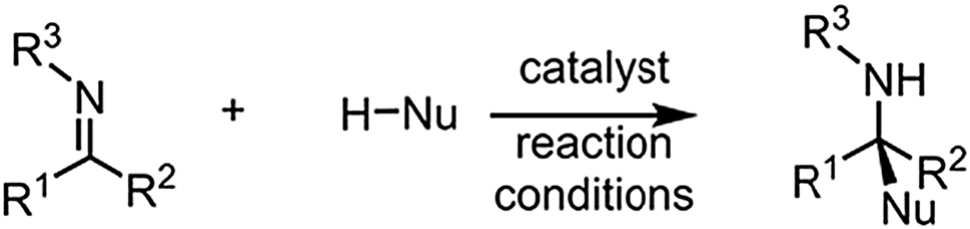
Generalized reaction scheme.

To provide the quantitative analysis, CPA reactions from various sources are collected by Reid et al.^2^. Specifically, the data set contains 342 CPA reactions from “the addition of protic nucleophiles to imines catalyzed by chiral 1,1’-bi-2-naphthol-(BINOL)-derived phosphoric acids bearing aromatic groups at the 3 and 3’    positions.” Figure 1 shows the generalized reaction scheme, where $\text {R}^{1}, \text {R}^{2}$,and $\text {R}^{3}$ are substituents, which summarize all the suitable substructures in the reactions. Nu indicates Nucleophile denoting an electron pair to form a chemical bond with another atom or molecule^15^.

**Figure 2:**
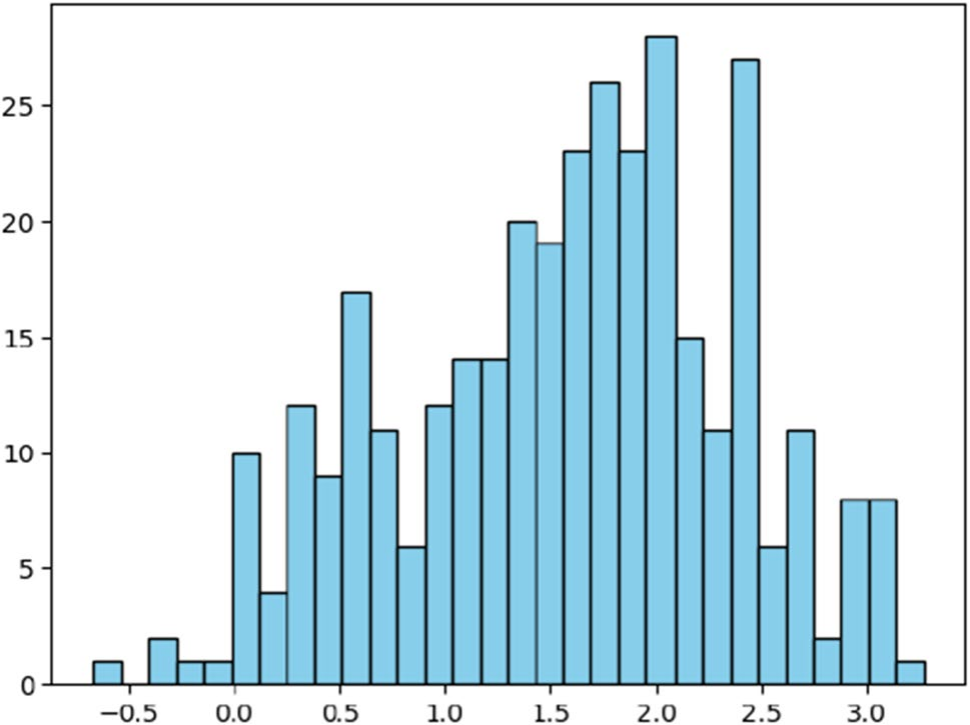
Histogram of enantioselectivity from 342 CPA reactions.

In addition, Terada et al.^16^, Chen et al.^17^, and Zahrt et al.^18^ offered 15, 15, and 34 reactions, respectively, from “addition of enecarbamates to benzoyl imines,” “hydrogenation of fluorinated alkynyl ketimines,” and “addition of thiols to imines.” Each reaction includes features describing the structure of molecules, including bond lengths, angle measurements, and molecular intensities^3^. Specifically, the numerical features of molecules were derived from density functional theory calculations and molecular topologies to describe solvents (160 features), catalysts (85 features), nucleophiles (15 features), and imines (22 features)^2^. Each reaction’s enantioselectivity ($\Delta \Delta \text {G}^{\ddag }$) is also collected. The goal is to build a robust model that predicts $\Delta \Delta \text {G}^{\ddag }$ of a reaction given the features of the catalyst, imine, nucleophile, and solvent. Specifically, 307 CPA reactions from Reid et al.^2^ are used as training data sets, and the remaining reactions are utilized as testing data sets. For the examination of outliers of enantioselectivity from 342 CPA reactions, we checked the normality of data samples, where Fig. 2 shows the histogram of enantioselectivity from 342 CPA reactions. In addition, the p-value of the Shapiro-Wilk test^19^, which shows that the null hypothesis represents the data drawn from a normal distribution, is 0.98. Therefore, the data follows a normal distribution. Since the data follows a normal distribution, we used z-scores of 342 samples to check the outliers. The minimum and maximum z-scores of all samples are -2.7 and 2.1, respectively, between -3 and 3, indicating that the dataset we used for the analysis has no outliers^20^.

## Methods and results

**Figure 3:**
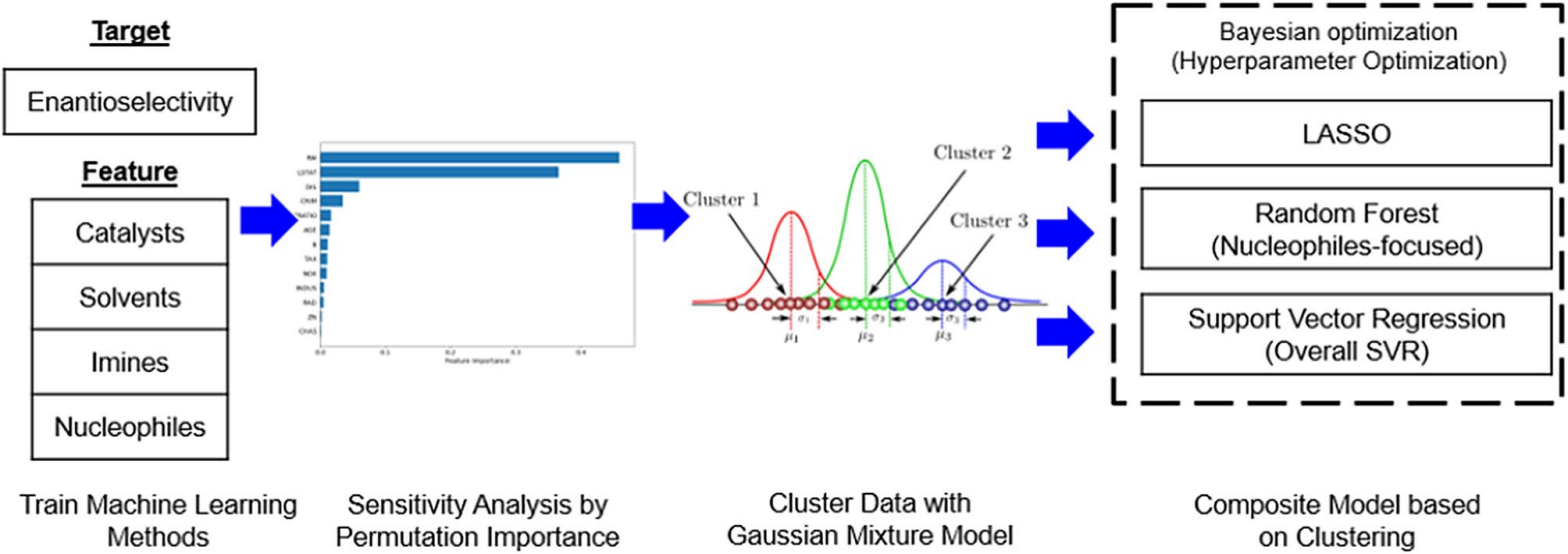
Overview of the proposed composite machine learning method.

Figure 3 shows the overview of the proposed method in this paper. To accurately predict the enantioselectivity of each reaction, several machine learning methods are trained with the training data. During the training procedure, the hyperparameters of each machine learning method are optimized based on Bayesian optimization. Furthermore, the representative sensitivity analysis called permutation importance calculates the informative features for predicting $\Delta \Delta \text {G}^{\ddag }$ in the training data. In the testing phase, the informative features from the preceding step are extracted from testing data. Then, the features are compared with those of the training data to cluster the testing data. Specifically, the Gaussian Mixture Model (GMM) trained with the features from training data clusters the testing data. The clustering results provide the appropriate regression method from the proposed composite machine learning method to the corresponding testing data.

Detailed descriptions of training and testing procedures are provided in the remaining section. Specifically, the illustrations of machine learning methods used in this paper are provided in “Section Machine learning method.” Subsequently, the regression analyses, including the Bayesian optimization and sensitivity analysis, are listed in “Sections Regression analysis and Performance evaluation.” Finally, the proposed composite machine learning method is demonstrated in “Section Composite model development and evaluation” with its efficacy in predicting enantioselectivity.

### Machine learning methods

In this paper, five widely used machine learning methods (i.e., LASSO^21^, Decision Tree^22^, Random Forest^23^, Gradient Boosting^24^, and Support Vector Regression^25^) are described. The methods are used for predicting the enantioselectivity of the reaction ($\Delta \Delta \text {G}^{\ddag }$) using features of imine, nucleophile, catalyst, and solvent.

- **LASSO**: Least absolute shrinkage and selection operator (LASSO)^21^ is a linear regression method that applies the shrinkage technique to encourage the coefficients of the regression model towards a zero. By utilizing $\ell _{1}$-regularization, LASSO achieves variable selection by penalizing coefficients based on their magnitude, resulting in coefficients being pushed closer to zero. Consequently, this penalty causes numerous coefficients to become zero, leaving only the variables strongly correlated with the response variable for prediction purposes.
- **Decision Tree**: Decision tree (DT) is a non-parametric method that employs straightforward decision rules for prediction purposes^22^. Specifically, the method divides data sets into smaller groups by utilizing a series of decision nodes, where the chosen path is determined by whether a specific condition is satisfied or not. DT is capable of capturing intricate and nonlinear relationships among features.
- **Random Forest**: Random forest (RF) is an ensemble learning method that constructs a collection of decision trees by training them on different subsets of features from the training dataset^23^. Combining these individual decision trees, the ensemble method effectively mitigates overfitting compared to a single decision tree. In RF, the prediction is determined by calculating the mean output of its decision trees.
- **Gradient Boosting**: Gradient Boosting (GB) is an additional ensemble learning method that creates a collection of decision trees to make predictions^24^. However, it differs from an RF, which constructs independent trees and averages their outcomes for predictions. Instead, GB utilizes the boosting technique^26^ to train and combine a sequence of trees to produce superior results compared to individual trees. Each additional tree introduced through the boosting technique is trained to minimize the residual error of the preceding tree. Furthermore, the GB training process assigns a weight to each tree. Finally, the ultimate prediction is determined by calculating the weighted average of the predictions made by the individual trees.
- **Support Vector Regression**: Support Vector Regression (SVR) is a machine learning method employed for regression analysis^25^. In contrast to support vector machines utilized for classification purposes, SVR endeavors to discover a hyperplane that most effectively fits the data points within a continuous space. Specifically, SVR identifies the hyperplane that maximizes the margin (distance) between the hyperplane and the nearest data samples while simultaneously minimizing prediction errors.

### Regression analysis

Regression analysis using the methods described in the “Machine learning method Section” is performed to predict the enantioselectivity given the features. The prediction capabilities are evaluated by using two performance measure, mean squared error (MSE) and $\text {R}^{2}$ value. In addition, Bayesian optimization is used to optimize the representative hyperparameters used by each machine learning method. Finally, sensitivity analysis is provided to diagnose the significant features in the prediction. Detailed descriptions of performance measures, hyperparameter optimization, and sensitivity analysis are explained below.

- **Performance Measures**: Mean squared error (MSE) quantifies the difference between actual observations and the values predicted by a regression method by calculating the average of the squares of the deviations between the predicted and actual values. Consequently, the MSE measures the accuracy or error of the model’s predictions relative to the actual observations. On the other hand, $\text {R}^{2}$, also known as the coefficient of determination, assesses the goodness of fit of a model. Specifically, $\text {R}^{2}$ measures the amount of variation in the data samples explained by the regression method. An $\text {R}^{2}$ value of 1 signifies a perfect fit of the regression predictions to the data. In addition, $\text {R}^{2}$ above 0.75 generally indicates that the corresponding regression method explains most of the variance of the data^27^. In this analysis, the training data is used for Monte Carlo cross-validation in each replication to randomly split as training and validation data with a ratio of four to one^28^. Specifically, a hundred replications are performed. Therefore, the average and standard deviation of MSE and $\text {R}^2$ of validation data from a hundred replications are provided as performance measures.
- **Hyperparameter Optimization**: In machine learning, hyperparameter optimization or tuning is the problem of choosing a set of optimal hyperparameters for a learning algorithm^29^. Bayesian optimization is often used to adjust the hyperparameters of a well-performing model on the validation data. Specifically, it searches global optimal hyperparameters by building a probabilistic model called the surrogate function. It repeatedly assesses a promising hyperparameter configuration based on the current surrogate function and updates the surrogate function for the subsequent configurations. In this study, the representative hyperparameters in each method are used for Bayesian optimization. Specifically, the hyperparameters related to overfitting and underfitting are optimized. For example, the hyperparameters that control the $\ell _{1}$ and $\ell _{2}$ regularization of LASSO (alpha) and SVR (C), respectively, are optimized. In addition, the minimum number of samples required to split an internal node in DT is optimized. Finally, the number of gradient-boosting iterations in GB and the number of trees in RF are optimized, respectively. The above hyperparameters are optimized in Bayesian optimization based on the performance of the MSE in the validation data, which are randomly sampled from the training data and consist of 20% of the training data.
- **Sensitivity Analysis**: Sensitivity analysis is provided to diagnose the significant features in the regression method. Specifically, the permutation feature importance test, the representative sensitivity analysis technique, is utilized. The concept of permutation feature importance refers to the reduction in a model’s score when the value of a single feature is randomly shuffled^30^. Breaking the connection between the feature and the response variable (i.e., $\Delta \Delta \text {G}^{\ddag }$ in this paper) allows us to gauge the extent to which the model relies on that particular feature. This approach is advantageous because it is independent of the specific model used and can be performed multiple times with various permutations of the feature. For the model score, the mean squared error of prediction is used. The mean and standard deviation from a hundred replications of the permutation feature importance test is provided as the measure.

### Performance evaluation

We evaluated the performance of the regression methods using all features, features excluding imine’s features (i.e., Nucleophile-focused methods), and features excluding nucleophile’s features (i.e., Imine-focused methods).

**Table 1:** Performance evaluation of regression methods using all features with their optimal hyperparameters from Bayesian optimization.

Models	MSE (STD)	$\text {R}^{2}$ (STD)	Optimal hyperparameter
LASSO	0.313 (0.06)	0.889 (0.02)	Alpha $=$ 0.016
DT	0.339 (0.15)	0.880 (0.06)	Min split $=$ 4
GB	0.221 (0.09)	0.922 (0.03)	Num iter $=$ 93
RF	0.210 (0.08)	0.925 (0.03)	Num trees $=$ 69
SVR	0.182 (0.04)	0.936 (0.02)	C $=$ 9.99

**Figure 4:**
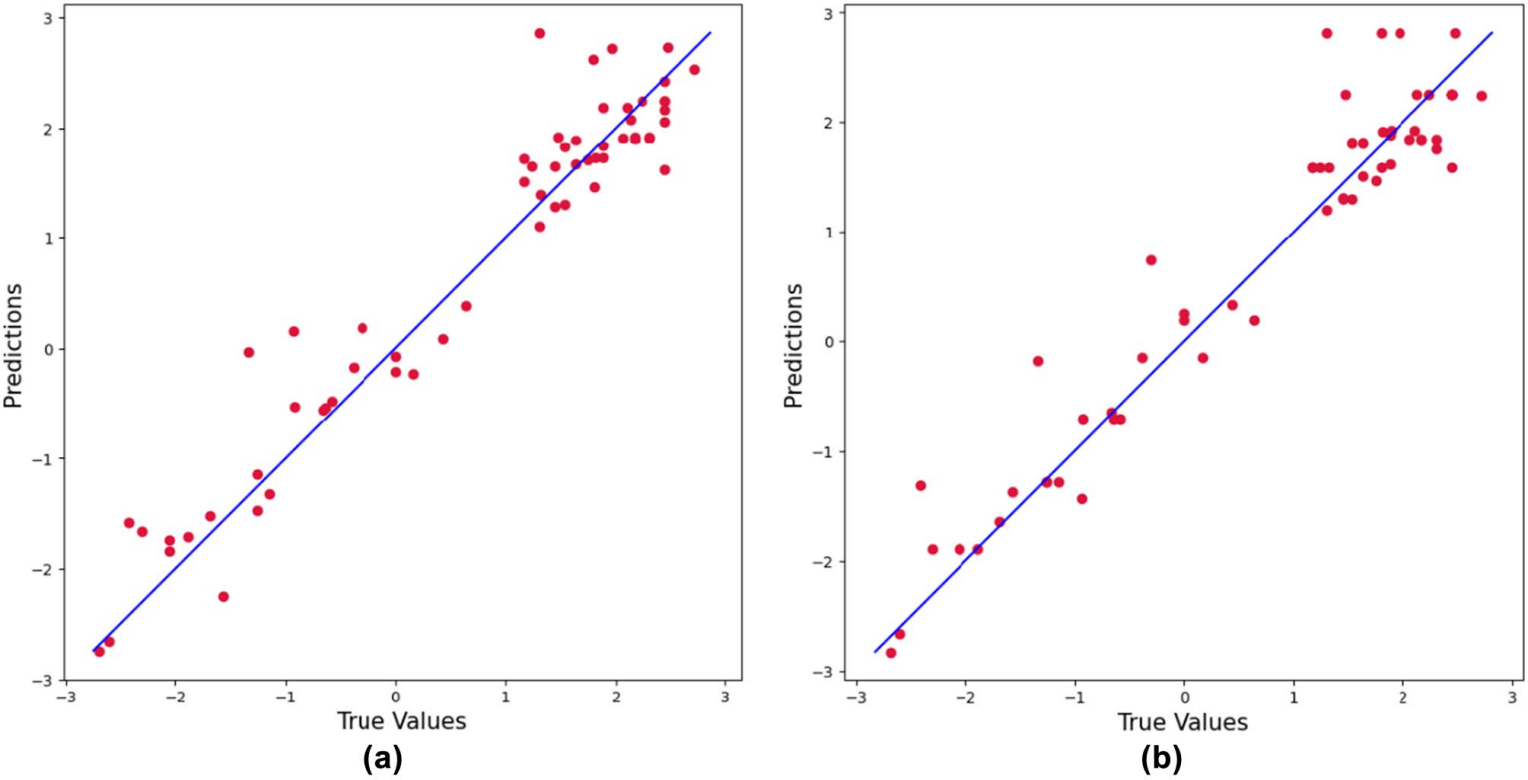
Scatter plot between actual $\Delta \Delta \text {G}^{\ddag }$ and predicted $\Delta \Delta \text {G}^{\ddag }$ from (**a**) SVR model trained with all features, (**b**) RF model trained without imine’s features. The blue line indicates the diagonal line.

**Figure 5:**
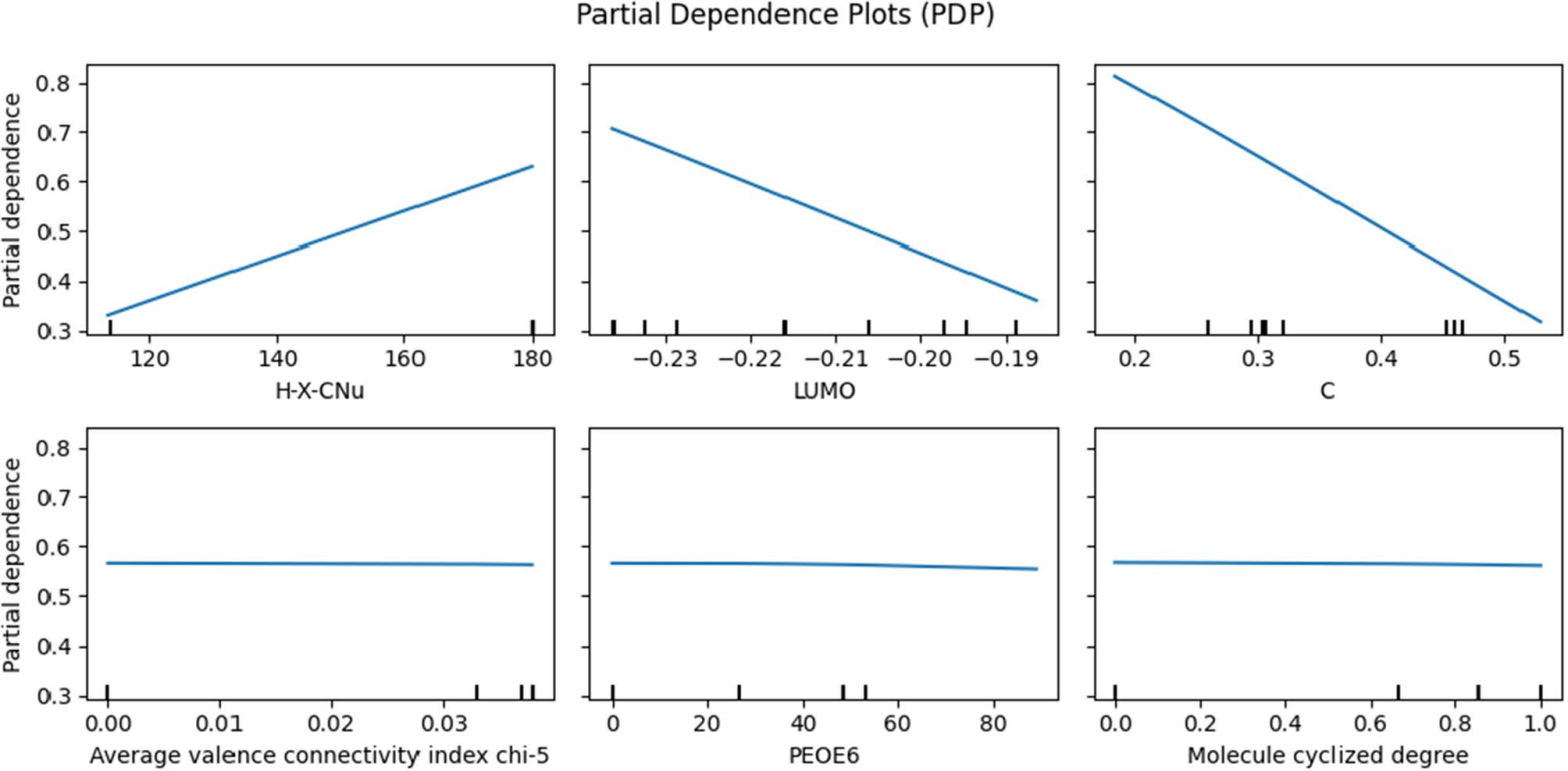
Partial dependence plots of the top three features with the highest and lowest permutation importance from SVR trained with all features.

- **Regression methods using all features**: Table 1 shows the prediction results using all features of imine, nucleophile, catalyst, and solvent. The results indicate that SVR performed the best, with an average $\text {R}^{2}$ value of 0.936 and the mean squared error (MSE) value of 0.182. SVR also achieves the least standard deviation (std) from 100 replications. The performance of SVR is significantly improved by hyperparameter optimization through Bayesian optimization. Specifically, the default value of the regularization parameter (C) in SVR in the sklearn package is one^31^. This setting achieves the average $\text {R}^{2}$ value of 0.916 with std 0.02 and the MSE value of 0.240 with std 0.05. Figure 4a shows the predictions match well with the ground truth, indicating the superior prediction capabilities of SVR. In addition, we can use two sample z-test^32,33^ between SVR and RF to show the statistical significance of the SVR. We can claim that the SVR of MSE and $\text {R}^{2}$ show a better performance than those of RF in 0.05 significance level since
  $$\begin{aligned} { \frac{0.182-0.210}{ \sqrt{\frac{(0.04)^{2}}{100} + \frac{(0.04)^{2}}{100}}} = -3.1305<-1.64=-Z_{0.05}, \quad \frac{0.936-0.925}{ \sqrt{\frac{(0.02)^{2}}{100} + \frac{(0.03)^{2}}{100}}} = 3.050851>1.64=Z_{0.05}.} \end{aligned}$$
  The results of the permutation feature importance test (see Table 2) show that the top eight features used by the SVR model include nucleophile’s features, including “H-X-CNu” and “H-X-Nu.” In addition, many inimine’s features, including “LUMO,” “C,” “N,” “SB1,” “iNH,” and “PG.” are included in the top features. Interestingly, the features of the catalyst and solvent have small impacts on the overall prediction. A possible explanation is that the training data lacks variations in catalysts and solvents^1^. Although imine gives strong indications of enantioselectivity prediction, it requires extra effort to obtain imine information. Specifically, imines are difficult to isolate and purify due to their sensitivity to hydrolysis^34^. Therefore, the regression models without using the imine features are further investigated in the following section. Among the eight features in Table 2, the first row of Fig. 5 provides the partial dependence plots (PDP) of three features achieving the highest permutation importance. In addition, the PDP of three features achieving the lowest permutation importance (i.e., solvents) are provided in the second row of Fig. 5. PDP shows the dependence between the target response (i.e., enantioselectivity) and an input feature of interest, marginalizing over the values of all other input features. Intuitively, we can interpret the partial dependence as the expected enantioselectivity as a function of the input features of interest^35^. The first row shows the partial dependence plots of “H-X-CNu,” “LUMO,” and “C.” The plots show that the features have a linear relationship with predicted enantioselectivity. Specifically, the ranges of predicted enantioselectivity from three features with the highest permutation importance are approximately 0.5, while those with the lowest permutation importance are approximately 0.01. The results demonstrate the significant impact of Imine and Nucleophile on the prediction of enantioselectivity in CPA reactions, while solvents do not (Fig. 5).

**Table 2:** The top eight most important features from the SVR model trained with all features.

Feature	Molecule	Mean	STD
H-X-CNu	Nucleophile	0.058	0.010
LUMO	Imine	0.055	0.011
C	Imine	0.039	0.008
H-X-Nu	Nucleophile	0.032	0.007
N	Imine	0.028	0.008
SB1	Imine	0.025	0.006
SubS	Imine	0.018	0.006
PG	Imine	0.017	0.006

The Feature column lists the feature names. The Molecule column lists the molecule category of each feature. The mean and standard deviation of the importance score are provided.

**Figure 6:**
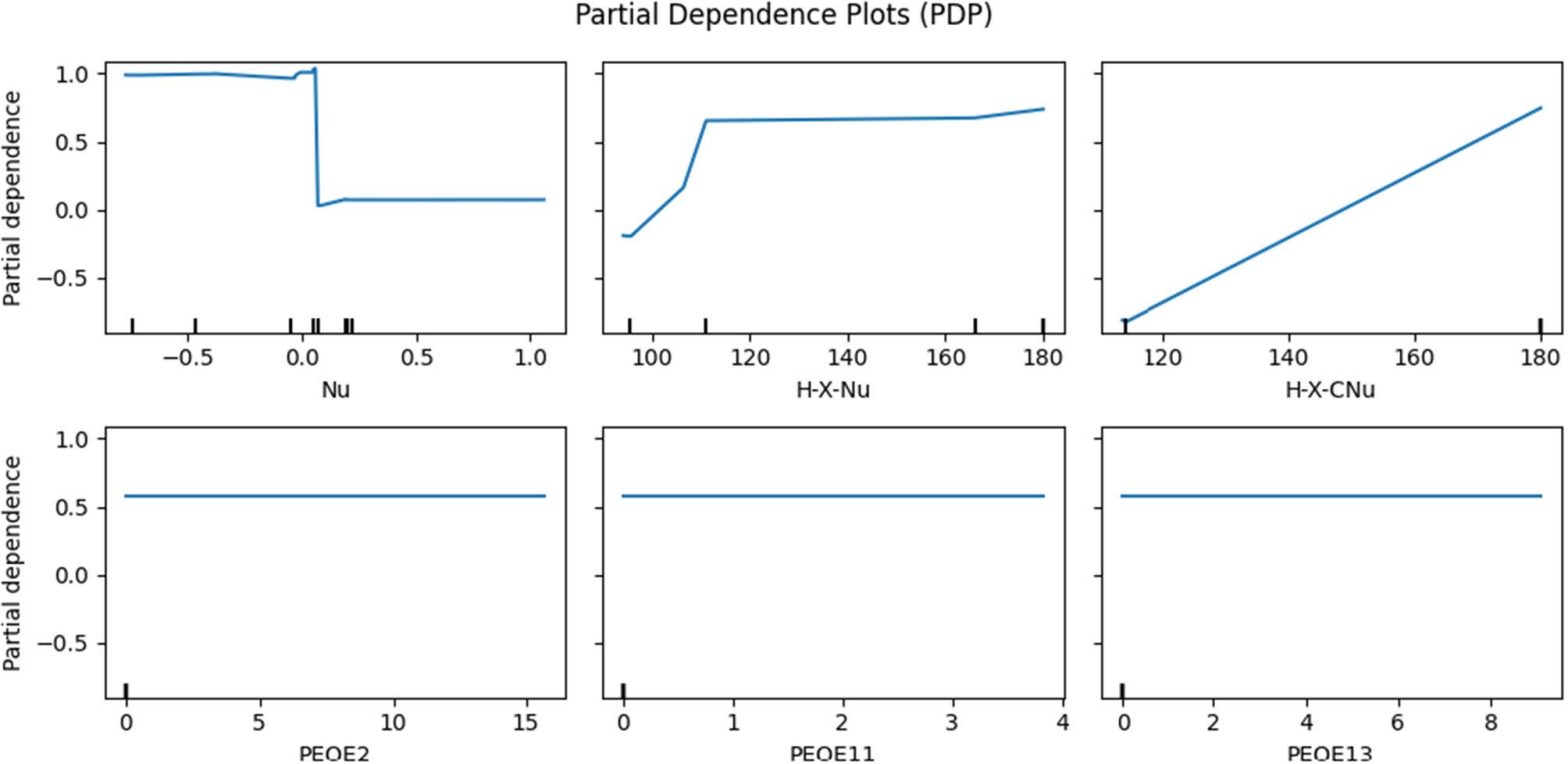
Partial dependence plots of the top three features with the highest and lowest permutation importance from RF trained without Imine’s features.

- **Nucleophile-focused models**: Table 3 compares the performance of regression methods without using the imine features. The results indicate that RF performed the best, with an average $\text {R}^{2}$ value of 0.932 and the MSE value of 0.192. RF also achieves the least std from 100 replications. Figure 4b illustrates the scatter plots of the RF, demonstrating the successful prediction results from RF. Similar to the previous section, we used two sample z-test between RF and SVR to show the statistical significance of the RF. We can claim that the RF of MSE and $\text {R}^{2}$ show a better performance than those of SVR in 0.05 significance level since
  $$\begin{aligned} {\frac{0.192-0.230}{ \sqrt{\frac{(0.05)^{2}}{100} + \frac{(0.06)^{2}}{100}}} = -4.8654<-1.64=-Z_{0.05}, \quad \frac{0.932-0.918}{ \sqrt{\frac{(0.02)^{2}}{100} + \frac{(0.02)^{2}}{100}}} = 4.9497>1.64=Z_{0.05}.} \end{aligned}$$
  Table 4 shows the results of the feature permutation test. Since there are no features from imine, the top eight features consist of the nucleophile, including “Nu,” “H-X-Nu,” “H-X-CNu,” “Polarizability,” “iXH,” “nXH,” “HOMO,” and “L.” Among the eight features in Table 4, the first row of Fig. 6 provides the PDP of three features achieving the highest permutation importance. In addition, the PDP of three features achieving the lowest permutation importance (i.e., solvents) are provided in the second row of Fig. 6. The first row shows the partial dependence plots of “Nu,” “H-X Nu,” and “H-X-CNu.” Specifically, the ranges of predicted enantioselectivity from three features with the highest permutation importance are varied from 0.8 to 1.6, while those with the lowest permutation importance are less than 0.01. The results demonstrate the significant impact of Nucleophile on the prediction of enantioselectivity in CPA reactions, while solvents do not.

**Table 3:** Performance evaluation of regression methods using all features excluding imine’s features with their optimal hyperparameters from Bayesian optimization.

Models	MSE (STD)	$\text {R}^{2}$ (STD)	Optimal hyperparameter
LASSO	0.628 (0.13)	0.778 (0.05)	Alpha $=$ 0.043
DT	0.259 (0.11)	0.908 (0.04)	Min split $=$ 8
GB	0.240 (0.06)	0.915 (0.02)	Num iter $=$ 76
RF	0.192 (0.05)	0.932 (0.02)	Num trees $=$ 28
SVR	0.230 (0.06)	0.918 (0.02)	C $=$ 9.99

**Table 4:** The top eight most important features from the RF model trained without imine’s features.

Feature	Molecule	Mean	STD
NU	Nucleophile	0.796	0.305
H-X-Nu	Nucleophile	0.406	0.176
H-X-CNu	Nucleophile	0.389	0.216
Polarizability	Nucleophile	0.143	0.249
iXH	Nucleophile	0.113	0.137
nXH	Nucleophile	0.017	0.016
HOMO	Nucleophile	0.014	0.009
L	Nucleophile	0.012	0.006

The Feature column lists the feature names. The Molecule column lists the molecule category of each feature. The mean and standard deviation of the importance score are provided.

- **Imine-focused models**: For the comparison, the performances of Imine-focused models trained without using nucleophile’s features are examined. Interestingly, the Imine-focused models did not perform quite as well as other models reported in the previous sections. Specifically, SVR achieves the best prediction results using the data without nucleophile’s features. However, its average MSE is above 0.3, and $\text {R}^{2}$ is less than 0.9, while the best regression model in Tables 1 and 3 achieves an average MSE of less than 0.2, and $\text {R}^{2}$ is higher than 0.9. Combined with the results in previous sections, it can be inferred that the imine’s features might be well explained by the other molecules involved in the same reactions, while nucleophile’s features are not. Because of relatively poor performance, Imine-focused models are not used as components in the following composite model.

### Composite model development and evaluation

Typically, the regression methods achieve noticeably better prediction results in the training data than in the testing data. This is the expected result since the data features from the training data and testing data are different. Therefore, the feature similarity between training and testing data must be investigated in advance. Specifically, imine and nucleophile play a significant role in predicting training data, as shown in Tables 2 and 4. Hence, it could be problematic when applying the best models in Tables 1 and 3 to new CPA reactions whose imine and/or nucleophile are very different from those in the training data (i.e., new samples could fall in the low-density regions of the training data)^1^.

One intriguing solution is to use multiple regression methods and combine them into a composite model^1^. Then, the appropriate regression model can be selected from the composite model according to the features of the testing data. Based on the performance evaluations from “Section Performance evaluation,” the following three prediction models are included in the proposed composite model: an SVR method trained by all features (overall SVR model in Table 1), an RF method trained without imine’s features (Nucleophile-focused RF model in Table 3), and a linear regression model trained via the LASSO algorithm using all features. The overall SVR method in Table 1 is able to make strong predictions when both the imine and nucleophile of testing data are similar to those in the training data. The Nucleophile-focused RF model in Table 3 would demonstrate robust predictive capability when the nucleophile’s features of testing data are similar to those in the training data while the imine’s features are not. In contrast, the LASSO model trained with all features is utilized if the nucleophile’s features are not similar. Based on the sensitivity analysis in “Section Performance evaluation”, it is evident that nucleophile plays the most significant role in the prediction of training data. In addition, nucleophile’s features are hardly expressed with other features compared to features of imine based on the analysis in “Section Performance evaluation”. Therefore, if nucleophile’s features are not similar between training and testing data, the existing model trained with training data would not be useful. In this case, LASSO, which is a simpler linear model than other machine learning methods in this paper, is chosen. This is because the complex model often leads to poor performance on unseen data, resulting in a lack of generalizability and, consequently, limited applicability of the model^36^.

**Figure 7:**
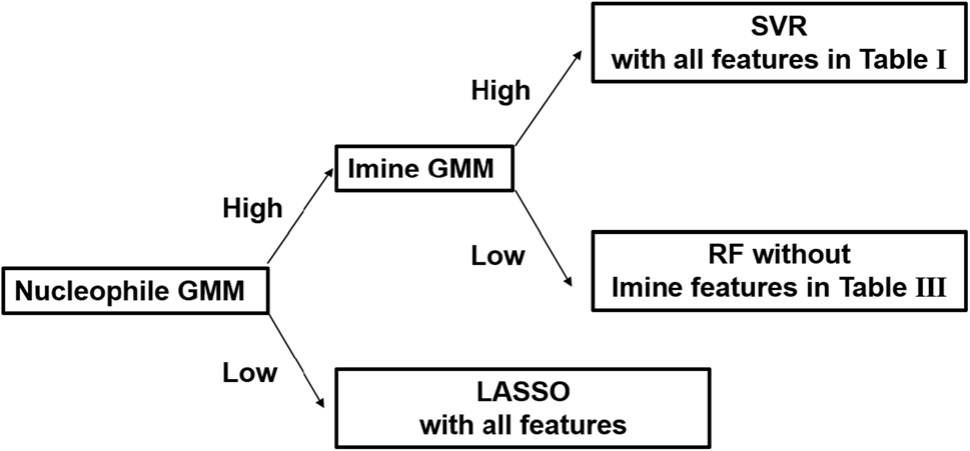
The architecture of the proposed composite model. One of three models is selected based on feature similarities from the GMM model to accurately predict the enantioselectivity of each reaction ($\Delta \Delta \text {G}^{\ddag }$).

**Figure 8:**
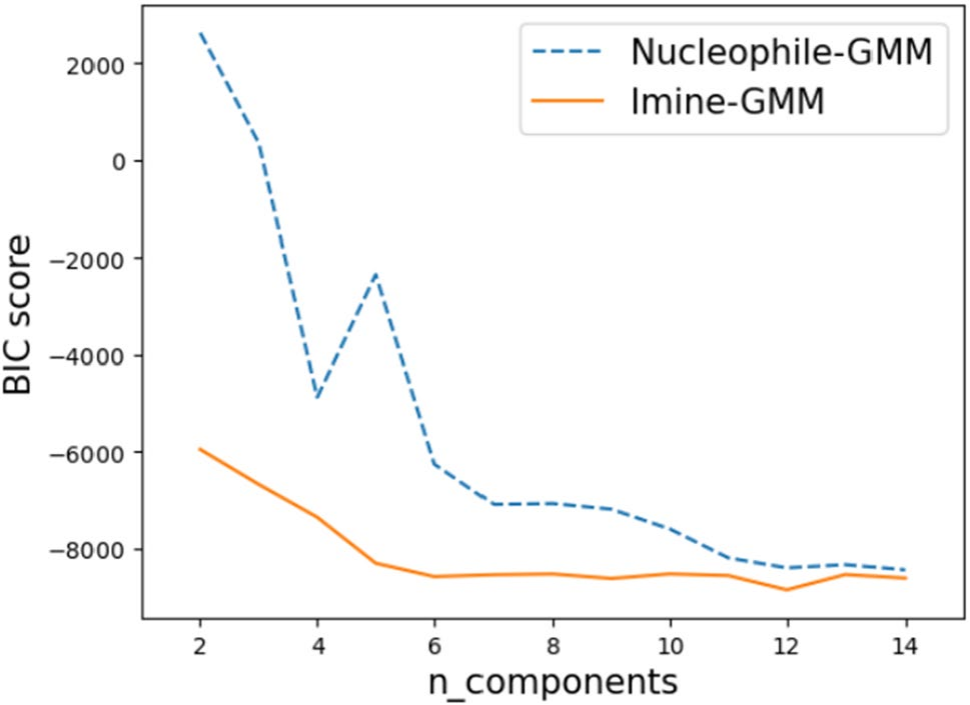
BIC value of Nucleophile-GMM and Imine-GMM by varying the number of Gaussian components.

**Figure 9:**
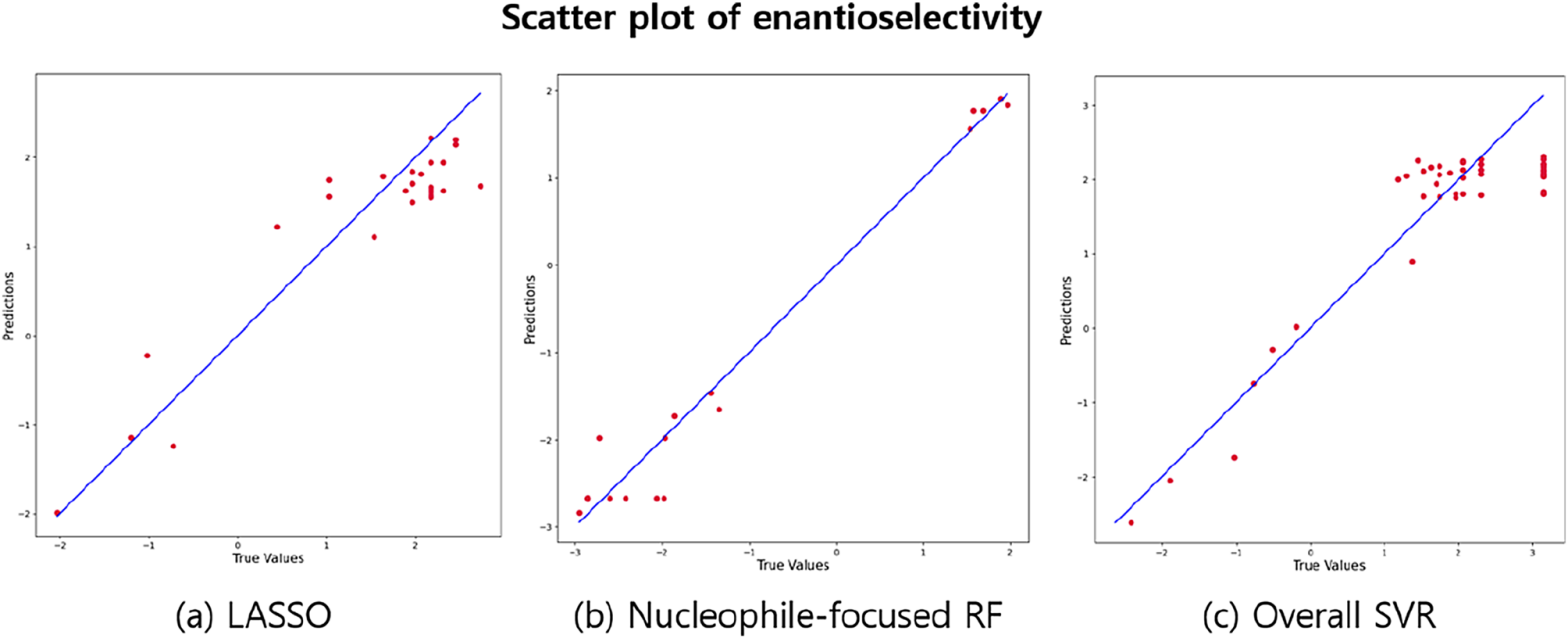
Scatter plot between actual $\Delta \Delta \text {G}^{\ddag }$ and predicted $\Delta \Delta \text {G}^{\ddag }$ by the regression methods from the proposed composite machine learning method, including (**a**) LASSO; (**b**) Nucleophile-focused RF; (**c**) Overall SVR.

- **Model Selection via Gaussian Mixture Model**: To systematically provide suitable regression methods based on the feature comparison between training data and testing data, the composite model whose architecture is shown in Fig. 7 is proposed. Specifically, the composite model compares the similarities of nucleophile and imine between training and testing data. To achieve this objective, the nucleophile and imine density functions from training data are respectively approximated by two Gaussian mixture models (GMM)^37^ via the Expectation-Maximization algorithm^38^. The Nucleophile-GMM approximates the joint distribution of important nucleophile’s features (H-X-Nu, H-X-CNu, Nu, and Polarizability) chosen as the top four important features by the Nucleophile-focused RF model in Table 4. Similarly, the Imine-GMM approximates the joint distribution of the imine’s features (C, N, SL, and LUMO) based on their importance in the overall SVR model listed in Table 2. GMM is defined as a linear combination of multiple Gaussian distributions. Therefore, the number of Gaussian distributions needs to be determined by the users. In the proposed composite model, the Bayesian information criterion (BIC)^39^ is used to determine the number of Gaussian components. When fitting models, it is possible to increase the maximum likelihood by adding parameters. However, it may result in overfitting. BIC attempts to resolve this problem by introducing a penalty term for the number of parameters in the model. The models with lower BIC are generally preferred. Based on the BIC value of Nucleophile and Imine-GMMs by varying the number of Gaussian components, as shown in Fig. 8, the Nucleophile and Imine-GMMs are selected to have 14 and 12 Gaussian components, respectively. After the Nucleophile and Imine-GMMs are fitted to training data, the average log-likelihood of the important features of nucleophile and imine in testing data are calculated from fitted GMMs. The value is considered high if it is greater than one. Otherwise low^1^.

- **Performance Evaluation**: As mentioned in “Section Data”, 35, 15, 15, and 34 CPA reactions are provided from various sources, including Reid et al.^2^, Terada et al.^16^, Chen et al.^17^, and Zahrt et al.^18^, respectively as testing data. To provide adequate cases regarding the feature similarities of nucleophile and imine between training and testing data, 35 CPA reactions from Reid et al.^2^ are randomly split into 12, 12, and 11 and provided to the reactions from Terada et al.^16^, Chen et al.^17^, and Zahrt et al.^18^, respectively. These three groups are denoted as reaction types R-A, R-B, and R-C, respectively, in Tables 5 and 6 as testing data. Table 5 shows the test results of the composite model. The first row in Table 5 lists three reaction types used in test data. The second row indicates the prediction model chosen by the composite model for the corresponding reaction types listed in the second, third, and fourth columns. The third row denotes the average log-likelihood of each reaction type concerning Imine-GMM, and the fourth row lists that with respect to Nucleophile-GMM. The third and fourth rows detail how the composite model chooses a method for each reaction type. Based on the criterion described in this section, all three test data sets are separated into each regression method. The $\text {R}^{2}$ of all three test data sets achieves higher than 0.75 from the proposed composite model, indicating that the proposed method successfully predicts the enantioselectivity in the test data set^27^ (Fig. 9). Unless the proposed composite model exists, users would reasonably select the SVR model trained with all features from Table 1. In addition, the users could simply use linear regression (LR)^2^. Therefore, the performances of the SVR model trained with all features and LR model are provided in Table 6 to show the effectiveness of the proposed composite machine learning method. Specifically, the forward step-wise LR method was employed for model development for LR method with 5-fold cross validation^2^. In addition, features that have significant similarities are automatically removed from LR, defined by 0.3 collinear criteria from Reid et al.^2^. The results show a much poorer performance than the proposed composite model’s performance described in Table 5. Therefore, it shows the effectiveness of the proposed composite model. Specifically, the results represent the significance of diagnosing the feature similarities and choosing the suitable model for accurate enantioselectivity prediction from the composite model.

**Table 5:** Performance evaluation of testing data using the proposed composite model based on the reactions.

Reaction types	R-A	R-B	R-C
Method chosen by the composite model	LASSO	Nucleophile-focused RF	Overall SVR
Average log-likelihood (Imine-GMM)	-885580	6.77	6.51
Average log-likelihood (Nucleophile-GMM)	13.95	-443.86	13.41
MSE	0.24	0.07	0.48
$\text {R}^{2}$	0.84	0.97	0.75
Optimal hyperparameter	Alpha $=$ 0.016	Num trees $=$ 28	C $=$ 9.99

**Table 6:** Performance evaluation of testing data using the SVR and LR^2^ trained with all features.

Reaction types	R-A		R-B		R-C	
Predictors	Overall SVR	LR^2^	Overall SVR	LR^2^	Overall SVR	LR^2^
MSE	0.85	0.64	0.31	0.72	0.48	0.67
$\text {R}^{2}$	0.45	0.58	0.89	0.75	0.75	0.64

## Conclusions

The objective of this study is to provide a framework for quantitatively analyzing the stereoselectivity of CPA reactions. Specifically, we have developed a novel composite machine learning model to accurately predict the enantioselectivity of any given CPA reaction, representing the degree to which one enantiomer is preferentially produced by the reaction. The inputs of our model are the features calculated from the molecules involved in reactions, including imine, nucleophile, solvent, and catalyst. The composite model uses GMMs to approximate the distributions of key features from nucleophile and imine, which allows the model to select more appropriate predictors and take advantage of the strengths of different machine learning models. Based on the composite model, the prediction performances are significantly improved as measured by both MSE and $\text {R}^{2}$. Specifically, the MSE decreases by more than 70% compared to the results of the previous state-of-the-art machine learning method without applying our composite model. In addition, the composite model significantly increases the $\text {R}^{2}$ so that the actual and predicted enantioselectivity of CPA reactions are highly correlated. Finally, the generalizability demonstrated by our approach to the test data indicates that our method can be used to explore other chemical reactions in future research. In addition, the users using the proposed composite model could check if the model’s accuracy improves by removing the features with low permutation importance.

## Data Availability

All data used for the analysis in this paper are uploaded at http://github.com/cjh7/enantioselectivity.

## Code availability

All codes used for the analysis in this paper are uploaded at http://github.com/cjh7/enantioselectivity.

## References

[1] Li, J. *et al*. Predicting the stereoselectivity of chemical transformations by machine learning. arXiv preprint arXiv:2110.05671 (2021).

[2] Reid JP, Sigman MS (2019). Holistic prediction of enantioselectivity in asymmetric catalysis. Nature.

[3] Nugent TC (2010). Chiral Amine Synthesis: Methods, Developments and Applications.

[4] Silverio DL (2013). Simple organic molecules as catalysts for enantioselective synthesis of amines and alcohols. Nature.

[5] Moon S, Chatterjee S, Seeberger PH, Gilmore K (2021). Predicting glycosylation stereoselectivity using machine learning. Chem. Sci..

[6] Yu X (2022). Prediction of enantioselectivity in thiol addition to imines catalyzed by chiral phosphoric acids. J. Phys. Org. Chem..

[7] Gao, B. et al. A machine learning model for predicting enantioselectivity in hypervalent iodine (iii) catalyzed asymmetric phenolic dearomatizations. *CCS Chem.* 1–14 (2024).

[8] Hoque A, Sunoj RB (2022). Deep learning for enantioselectivity predictions in catalytic asymmetric $$\beta $$-c-h bond activation reactions. Digital Discov..

[9] Hong, Y., Welch, C. J., Piras, P. & Tang, H. Enhanced structure-based prediction of chiral stationary phases for chromatographic enantioseparation from 3D molecular conformations. *Analytical Chem.* (2024). 10.1021/acs.analchem.3c04028 38308813

[10] Ferraz-Caetano J, Teixeira F, Cordeiro MND (2024). Explainable supervised machine learning model to predict solvation gibbs energy. J. Chem. Inf. Model..

[11] Ward L (2021). Graph-based approaches for predicting solvation energy in multiple solvents: open datasets and machine learning models. J. Phys. Chem. A.

[12] Low K, Coote ML, Izgorodina EI (2022). Explainable solvation free energy prediction combining graph neural networks with chemical intuition. J. Chem. Inf. Model..

[13] Lim H, Jung Y (2021). MLSolvA: Solvation free energy prediction from pairwise atomistic interactions by machine learning. J. Cheminform..

[14] Pathak Y, Mehta S, Priyakumar UD (2021). Learning atomic interactions through solvation free energy prediction using graph neural networks. J. Chem. Inf. Model..

[15] Solomons TG, Fryhle CB (2008). Organic Chemistry.

[16] Terada M, Machioka K, Sorimachi K (2006). High substrate/catalyst organocatalysis by a chiral brønsted acid for an enantioselective aza-ene-type reaction. Angew. Chem. Int. Ed..

[17] Chen M-W (2018). Organocatalytic asymmetric reduction of fluorinated alkynyl ketimines. J. Org. Chem..

[18] Zahrt AF (2019). Prediction of higher-selectivity catalysts by computer-driven workflow and machine learning. Science.

[19] Dudley, R. The Shapiro–Wilk test for normality (2023).

[20] Stevens JP (2013). Intermediate Statistics: A Modern Approach.

[21] Tibshirani R (1996). Regression shrinkage and selection via the lasso. J. R. Stat. Soc. Ser. B Stat Methodol..

[22] Loh W-Y (2011). Classification and regression trees. Wiley Interdiscipl. Rev. Data Mining Knowl. Discov..

[23] Breiman L (2001). Random forests. Mach. Learn..

[24] Drucker, H. Improving regressors using boosting techniques. In *Icml*, vol. 97, 107–115 (Citeseer, 1997).

[25] Smola AJ, Schölkopf B (2004). A tutorial on support vector regression. Stat. Comput..

[26] Schapire RE (1990). The strength of weak learnability. Mach. Learn..

[27] Tsiambaos G, Sabatakakis N (2004). Considerations on strength of intact sedimentary rocks. Eng. Geol..

[28] Xu Q-S, Liang Y-Z (2001). Monte Carlo cross validation. Chemom. Intell. Lab. Syst..

[29] Frazier, P. I. A tutorial on Bayesian optimization. arXiv preprint arXiv:1807.02811 (2018).

[30] Kaneko H (2022). Cross-validated permutation feature importance considering correlation between features. Anal. Sci. Adv..

[31] scikitlearn. sklearn.svm.svc. https://scikit-learn.org/stable/modules/generated/sklearn.svm.SVC.html.

[32] Zimmerman, D. W. Correcting two-sample “z” and “t” tests for correlation: An alternative to one-sample tests on difference scores. *Psicologica Int. J. Methodol. Exp. Psychol.* **33**, 391–418 (2012).

[33] Hogg, R. V., Tanis, E. A. & Zimmerman, D. L. *Probability and Statistical Inference*, vol. 993 (Macmillan, 1977).

[34] Walker, M. A. Libretexts. https://chem.libretexts.org.

[35] Shi H, Yang N, Yang X, Tang H (2023). Clarifying relationship between pm2.5 concentrations and spatiotemporal predictors using multi-way partial dependence plots. Remote Sens..

[36] Buchanan R, Whiting R, Damert W (1997). When is simple good enough: a comparison of the Gompertz, Baranyi, and three-phase linear models for fitting bacterial growth curves. Food Microbiol..

[37] McLachlan, G. J. & Basford, K. E. *Mixture Models: Inference and Applications to Clustering*, vol. 38 (M. Dekker, 1988).

[38] Dempster AP, Laird NM, Rubin DB (1977). Maximum likelihood from incomplete data via the em algorithm. J. Roy. Stat. Soc. Ser. B (Methodol.).

[39] Neath AA, Cavanaugh JE (2012). The Bayesian information criterion: Background, derivation, and applications. Wiley Interdiscipl. Rev. Comput. Stat..

